# Pediatric physeal repair: from immune–angiogenic–osteogenic coupling to zonal biomimetic scaffolds

**DOI:** 10.3389/fcell.2026.1802968

**Published:** 2026-07-28

**Authors:** Yan-Hong Wang, Qin Hu, Dan Ma, Qiu Wang, Hui Zhou

**Affiliations:** Department of Rehabilitation Medicine, Key Laboratory of Birth Defects and Related Diseases of Women and Children, West China Second University Hospital of Sichuan University (WCSUH-SCU), Chengdu, Sichuan, China

**Keywords:** 3D printing, biomaterials, growth plate, hydrogel, interface engineering, physeal injury

## Abstract

Physeal injury in children can trigger bone bridge formation that arrests growth and leads to limb-length discrepancy, angular deformity, or both. Clinical bar resection with interposition reduces recurrence yet often fails to restore zonal growth-plate function. This mini review synthesizes recent bioengineering strategies that aim to prevent re-bridging while re-establishing a cartilage-permissive niche capable of supporting longitudinal growth. We focus on three platform classes that provide strong leverage for spatiotemporal control. Injectable viscoelastic hydrogels can be tuned in degradation, transport, and stress relaxation and can serve as local depots for staged factor delivery. Deterministic printed architectures prescribe geometry and invasion routes and can be combined with bioactive compartments for hybrid control. Multiphasic composites introduce interfacial gating zones that target the defining failure mode of physeal repair, namely, mineralized continuity spanning epiphysis and metaphysis. To move beyond material taxonomies, we interpret the field through four coupled and engineerable knobs: immune response, vascular invasion, osteogenic takeover with bridge continuity, and time-dependent mechanics with viscoelasticity. Across platforms, durable success is most consistently associated with early control of vascular and osteogenic access to the defect core, preservation of a diffusion-limited cartilage niche, explicit interface gating, and longitudinal functional evaluation that captures growth rather than short-term histology alone.

## 1 Introduction

Physeal injuries are uniquely consequential in children because the growth plate is a zonally organized developmental organ that continuously drives longitudinal bone growth rather than acting as a passive cartilage filler Ağırdil (2020). Even small defects can initiate a physeal bar, a bony bridge that produces focal growth arrest and can evolve into limb-length discrepancy, angular deformity, or both. Current management typically relies on bar resection followed by interposition. Although this approach can reduce recurrence, it frequently fails to reconstitute native zonal architecture or durable growth potential Chung et al. (2011).

These clinical limitations define a distinct bioengineering problem. A successful construct must preserve resting and proliferative phenotypes that sustain column formation while restraining premature vascular invasion and osteogenic takeover, a common route to re-bridging. Scaffold-based strategies are appealing because they can impose spatially patterned microenvironments and interfaces, provide programmable biochemical conditioning through extracellular-matrix-mimetic cues and controlled factor delivery, and enable tuning of mechanics and transport properties such as viscoelasticity, permeability, degradation, and nutrient diffusion Ağırdil (2020), Wu et al. (2024). A persistent pitfall is direct translation of adult osteochondral repair logic, where vascularized subchondral integration is often desirable. In physeal repair, the same permissiveness can accelerate failure by enabling early vascular and osteogenic access to the defect core Chung et al. (2011), Wu et al. (2024).

Recent *in vivo* studies illustrate both the core tension and a practical path forward. Coupled tuning of hydrogel degradation and transport can shift outcomes toward cartilage-like repair rather than bar formation, and staged delivery that delays angiogenesis early while supporting chondrogenesis later can reduce bridging in rabbit models Erickson et al. (2020), Qiang et al. (2023). On this basis, we focus on three engineering platforms with strong spatiotemporal leverage: injectable viscoelastic hydrogels, printed scaffolds that prescribe geometry and invasion routes, and multiphasic composites incorporating interfacial gating zones Erickson et al. (2020), Wang et al. (2023), Yu et al. (2022).

**FIGURE 1:**
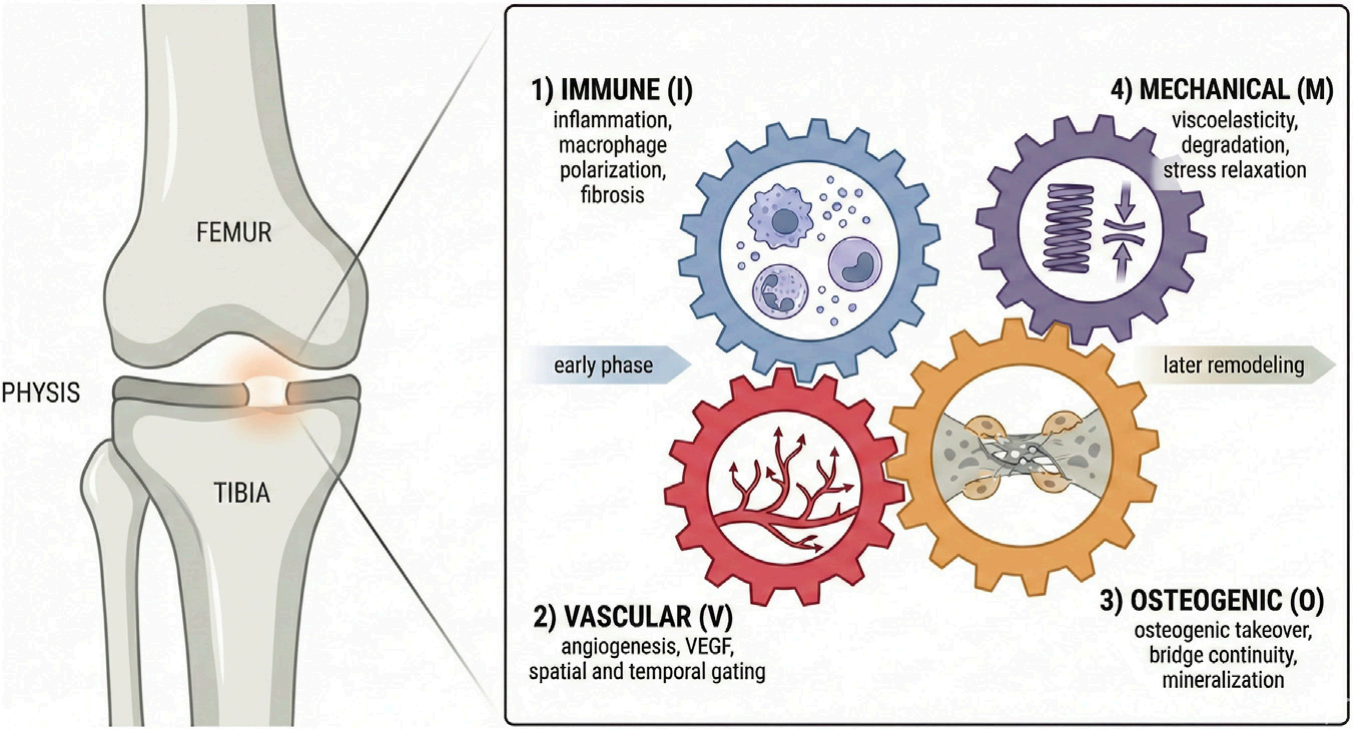
Four-knob framework for post-injury physeal repair. Left, a schematic of a pediatric knee showing a focal physeal defect at the growth plate; Right, an enlarged inset depicts the repair microenvironment as four coupled, engineerable processes represented by interlocking gears: immune response (I), vascular invasion (V), osteogenic takeover (O), and mechanics and viscoelasticity (M). The four knobs represent coupled, engineerable control axes that jointly determine whether a physeal defect remains cartilage-permissive or converts into a spanning mineralized bridge.

**FIGURE 2:**
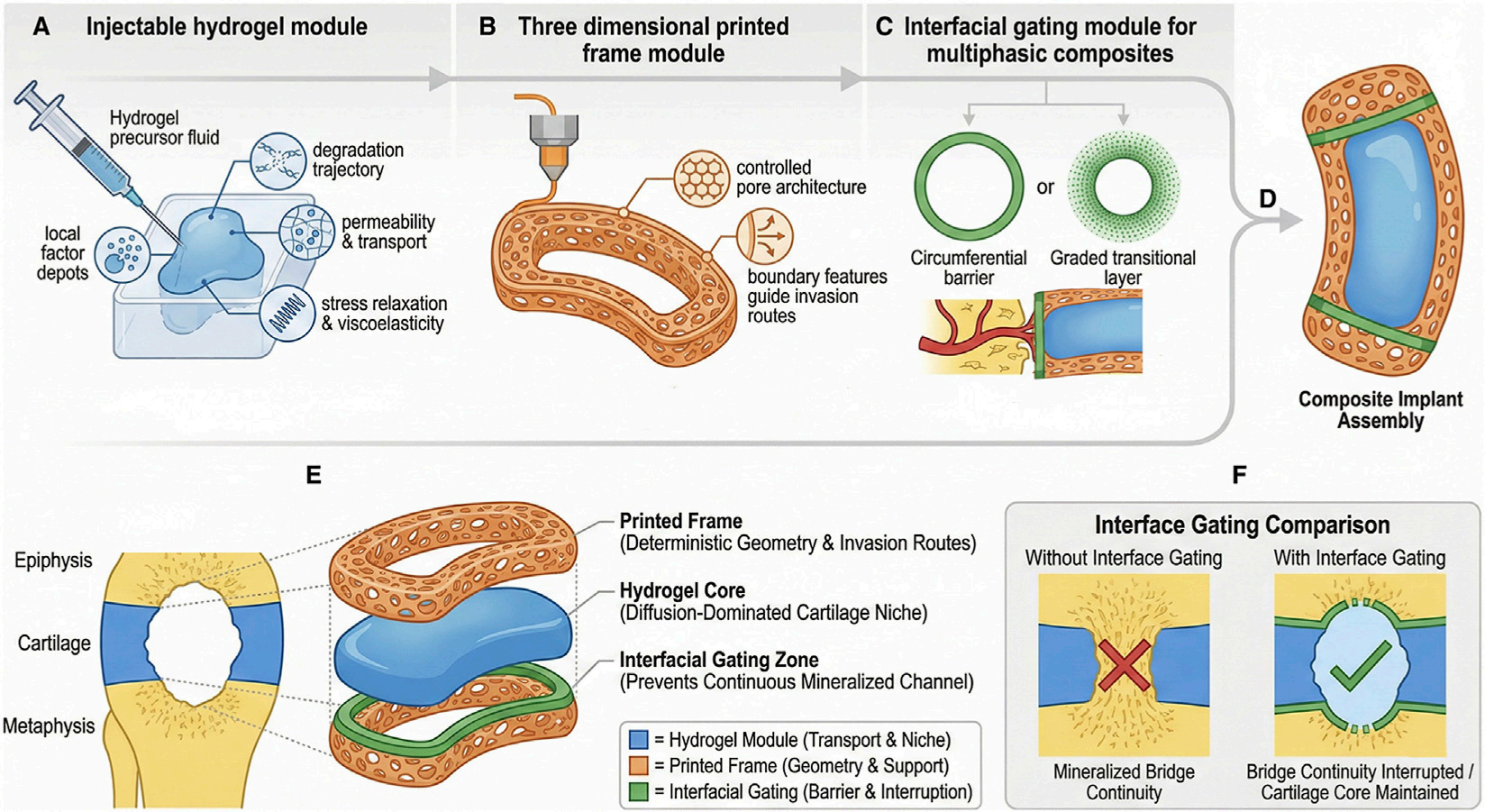
Modular design and fabrication logic for physeal repair constructs: **(A)** The injectable hydrogel module conforms to irregular defects and is tuned through time-dependent degradation, permeability, and viscoelastic stress relaxation, while also serving as a localized depot for staged cue presentation; **(B)** The three-dimensional printed frame module provides defect-matched geometry, anisotropy, and prescribed pore architecture that stabilizes the repair space and constrains invasion routes; **(C)** The multiphasic interface module introduces an interfacial gating zone, implemented as a circumferential barrier, a graded transition layer, or their combination, to limit early vascular entry and to prevent formation of a continuous mineralized channel across epiphysis and metaphysis; **(D)** Composite implant assembly; **(E)** Compartmentalized implant with printed frame maintains boundary conditions, the hydrogel core supports a diffusion-dominated cartilage-permissive niche, and the gating interface interrupts mineralized continuity while permitting peripheral integration; **(F)** A comparison inset highlights the key failure mode of spanning mineralized bridging when interface control is absent and the desired outcome of continuity interruption with a preserved cartilage-like core when interfacial gating is present.

Rather than cataloging materials, we use a four-knob framework—immune response, vascular invasion, osteogenic bridge continuity, and time-dependent mechanics with viscoelasticity—to connect platform design with dominant failure modes and control opportunities in physeal repair Yu et al. (2022), Fan et al. (2023), Wang et al. (2025), Zhang et al. (2026). This framing emphasizes that success requires not only cartilage-like tissue formation, but also prevention of a spanning mineralized bridge that compromises longitudinal growth. Accordingly, we highlight longitudinal growth and bar continuity as key translational readouts. The conceptual organization is summarized in Figure 1, and the corresponding modular scaffold design logic is illustrated in Figure 2.

## 2 Growth plate physiology underpinning longitudinal growth

Longitudinal growth emerges from endochondral ossification at the physis, where chondrocytes are organized into ordered compartments that couple cell-state progression to matrix turnover and a regulated vascular front Kronenberg (2003), Prein et al. (2016), Gerber et al. (1999), Hunter and Arsenault (1990). Resting-zone progenitors sustain the proliferative columnar engine, proliferative chondrocytes expand and orient the template, and hypertrophic chondrocytes remodel matrix while instructing vascular and osteogenic invasion near the metaphyseal junction Kronenberg (2003), Gerber et al. (1999), Abad et al. (2002), Mizuhashi et al. (2018). These programs are paced by intersecting paracrine networks that include Ihh–PTHrP signaling and additional pathways such as BMP, Wnt, FGF, and Notch, and are modulated by endocrine inputs including GH with IGF-1, thyroid hormone, glucocorticoids, and sex steroids Kronenberg and Chung (2001), Lui et al. (2014), Ornitz and Marie (2002), Mead and Yutzey (2009), Nilsson et al. (2005). Recent human growth-plate niche profiling further emphasizes that physical and chemical microenvironmental gradients are polarized across the physis, making species-aware reconstruction of zonal cues an important translational benchmark Xie et al. (2025).

Zonal identity is actively maintained. Lineage tracing identifies PTHrP-positive resting chondrocytes as a progenitor-like source for the proliferative compartment over extended timescales, and niche signals such as a Wnt-inhibitory environment help preserve slow-cycling behavior while enabling transition into column-forming progeny Mizuhashi et al. (2018), Hallett et al. (2021a). Hypertrophic differentiation is a coordinated transition that includes metabolic shifts, matrix specialization, and secretion of coupling factors that orchestrate the cartilage-to-bone transition Gerber et al. (1999), Kozhemyakina et al. (2015). Genetic lineage tracing further indicates that a fraction of hypertrophic chondrocytes can contribute to osteoblast and osteocyte lineages during endochondral bone formation and postnatal growth, supporting a tighter coupling between chondrogenic and osteogenic lineages than classical models implied Yang et al. (2014), Zhou et al. (2014), Hallett et al. (2021b).

Growth-plate function is also constrained by transport and vascular choreography. Interior cartilage is physiologically hypoxic, and HIF-1 α is required for chondrocyte survival and appropriate growth under low oxygen tension Schipani et al. (2001). At the hypertrophic front, VEGF from hypertrophic chondrocytes couples cartilage remodeling with angiogenesis and ossification, linking vascular access to endochondral progression Gerber et al. (1999). Normal physiology restricts where and when vessels enter, so disruption of this choreography by injury or inflammation can accelerate osteogenic replacement Hunter and Arsenault (1990), Fischerauer et al. (2011), Chung et al. (2014). Finally, postnatal growth slows as the growth plate undergoes a local program of senescence characterized by declining proliferation and structural change, a tempo that can be modulated by endocrine and pathophysiologic states Nilsson and Baron (2004), Lui et al. (2011), Marino et al. (2008), Weise et al. (2001).

## 3 Post-injury pathobiology translated into four controllable knobs

After physeal injury, healing often follows an ossification-skewed trajectory rather than restoring stable zonal cartilage, progressing through inflammatory, fibrogenic, osteogenic, and remodeling phases Zhou et al. (2004), Chung et al. (2006). Early immune dynamics, frequently dominated by acute inflammation, can bias downstream trajectories toward chondrogenic repair or fibro-osteogenic replacement Zhou et al. (2004), Chung et al. (2006). Angiogenesis is a central inflection point. Vascular invasion can be detected early and is repeatedly implicated as an upstream driver of bar formation through VEGF-mediated signaling Fischerauer et al. (2011), Chung et al. (2014). These features can be operationalized as four coupled knobs whose staged and spatially explicit control helps determine whether a defect remains cartilage-permissive or converts into a mineralized bridge: immune response, vascular invasion, osteogenic takeover with bridge continuity, and mechanics with viscoelasticity. Figure 1 should therefore be read as a coupling map rather than a linear pathway: inflammatory signals can open vascular routes, vascular access can accelerate osteogenic replacement, mineralized continuity can alter local mechanics, and altered load transfer can further bias cell fate and matrix remodeling.

### 3.1 Immune knob

The immune knob should first be understood as the injury-sensing axis of the damaged physis. Matrix disruption, chondrocyte death or stress, local hypoxia, and bleeding expose damage-associated signals to resident and recruited innate immune cells. Experimental growth-plate injury studies indicate that repair commonly proceeds through inflammatory, fibrogenic, osteogenic, and bone-bridge maturation phases rather than direct restoration of organized physeal cartilage Zhou et al. (2004), Chung et al. (2006), Chung et al. (2011), Chung and Xian (2014). During this sequence, early TNF- α , IL-1 β , IL-6, COX-2, and iNOS activity overlaps with fibrogenic and osteogenic signals such as PDGF-BB, FGF-2, Runx2, ALP, and osteocalcin, meaning that inflammation helps set the trajectory toward either cartilage-permissive repair or fibro-osseous bridging Zhou et al. (2004), Chung et al. (2006), Tong et al. (2022).

This response is useful only if it remains transient. Early inflammation supports debris clearance and reparative-cell recruitment, but persistent cytokine exposure can directly impair growth-plate cartilage programs. IL-1 β and TNF- α restrict recovery of growth-plate chondrogenesis and longitudinal bone growth, while IL-1 β can disturb chondrocyte maturation by downregulating FGFR-3 and p21, reducing proteoglycan synthesis, and decreasing Sox9 and type II collagen expression Tong et al. (2022), MacRae et al. (2006), Simsa-Maziel and Monsonego-Ornan (2012). Inflammation also couples to the other knobs: inflammatory and macrophage-derived factors can amplify VEGF-related angiogenic activity, fibrogenic tissue can become a substrate for vascular entry, and IL-6–SHP2-related signaling has been implicated in osteoclast differentiation after growth-plate injury Longoni et al. (2018), Newman et al. (2021), Zhang et al. (2025b). Thus, unresolved inflammation can suppress SOX9/COL2A1/aggrecan-rich repair while favoring matrix catabolism, vascular penetration, osteoclast activity, and mineralized bridging.

Recent immunomodulatory strategies provide useful but still incomplete proof of concept. MSC exosomes improved physeal repair and reduced limb-length discrepancy in young rats, yet did not fully eliminate bridge formation, suggesting that immune modulation must be coupled with vascular and osteogenic gating Wong et al. (2022). Exosome-loaded extracellular-matrix-mimic hydrogels and injectable photocrosslinkable acellular cartilage matrix hydrogels localize vesicle presentation and combine anti-inflammatory signaling with cartilage-derived matrix cues Guan et al. (2022), Si et al. (2024). Related cartilage and osteochondral systems, including IL-4-loaded decellularized cartilage matrices and ROS-responsive hydrogel/PLGA hybrids, are best viewed as mechanistic analogues that show how pro-resolving macrophage cues or oxidative-stress control can be embedded in matrices, rather than as direct physeal proof Tian et al. (2021), Wu et al. (2021).

### 3.2 Vascular knob

The vascular knob should first be understood as a spatial-control axis. In the intact physis, most cartilage is avascular and diffusion-dependent, whereas vessels are restricted to perichondrial and metaphyseal regions. VEGF from hypertrophic chondrocytes normally couples matrix remodeling with angiogenesis and ossification at the metaphyseal front, but injury can redirect this program into the defect core Gerber et al. (1999), Hunter and Arsenault (1990), Fischerauer et al. (2011). Once vessels enter the injured physis, they can carry endothelial cells, perivascular stromal cells, osteoclast-lineage cells, oxygen, and osteogenic cues into a niche that should remain cartilage-permissive; vascular invasion therefore becomes a gateway linking inflammation to mineralized bridge formation.

The therapeutic goal is not global angiogenic blockade, but local and time-limited vascular delay. Normal growth requires metaphyseal vascular activity, and systemic VEGF inhibition can reduce bony repair yet worsen growth impairment Chung et al. (2014). Local anti-VEGF delivery in rats reduced vascularization and bony bar formation while increasing cartilage-like repair tissue, supporting local vascular gating as a more appropriate strategy Erickson et al. (2021). Sequential release systems refine this principle: early bevacizumab release followed by later IGF-1 support in a GelMA-based rabbit model tested whether transient vascular delay can be paired with subsequent chondrogenic support rather than sustained anti-angiogenic suppression Qiang et al. (2023).

Architecture can serve the same knob by controlling routes of entry. Printed frames, compartmentalized implants, and interfacial barriers can limit the chance that vessels form a continuous epiphyseal–metaphyseal channel without requiring global angiogenic suppression. Osteochondral interface work, including printed scaffolds with calcified interfacial layers, further supports the concept that compact boundary layers can preserve compartmentalization and resist vascular entry from bone margins Wu et al. (2024), Wang et al. (2022), Nordberg et al. (2024a). Together, the contrast between systemic VEGF inhibition, local anti-VEGF delivery, sequential bevacizumab–IGF-1 release, and physical interface gating supports vascular control as a localized timing-and-routing problem rather than a simple anti-angiogenic endpoint Qiang et al. (2023), Chung et al. (2014), Erickson et al. (2021).

### 3.3 Osteogenic knob

The osteogenic knob should first be understood as a continuity-control axis. Bone bridge formation is not merely excess mineralized tissue; it is a connected mineralized conduit spanning epiphysis and metaphysis. After injury, inflammatory and fibrogenic repair can provide a provisional matrix that is invaded by vessels and osteogenic cells. If hypertrophic, osteoblastic, and mineralizing programs occupy the defect core, the result can be a bar that mechanically tethers longitudinal growth.

Osteogenic control therefore differs from nonspecific suppression of bone formation. Endochondral ossification is required at the metaphyseal front, but it becomes pathological when osteogenic differentiation and mineral deposition enter the defect core or connect both sides of the physis. BMP, IHH/PTHrP, Wnt/ β -catenin, TGF- β , and IGF-1 signaling all participate in growth-plate maintenance, chondrogenesis, hypertrophy, and osteogenesis, so pathway modulation must be interpreted by dose, location, and timing Lui et al. (2014), Kozhemyakina et al. (2015), Samsa et al. (2017), Long and Ornitz (2013). Enhanced BMP signaling in injured rat growth plates provides a useful cautionary example: a pathway that supports skeletal repair can still promote cartilage dysrepair when spatially or temporally inappropriate Su et al. (2021).

Recent strategies illustrate both molecular and structural approaches to this knob. Sustained MAPK14-targeting siRNA release from polyelectrolyte-complex hydrogels reduced osteogenic gene expression in MSCs *in vitro*, although the tested *in vivo* dose did not significantly reduce bony bar formation, highlighting the need for stronger spatial and temporal control Adhikari et al. (2024). Factor-delivering hydrogel/polymer scaffolds, such as PTH(1–34)-loaded PLGA microspheres combined with BMSCs, GelMA, and a PCL printed frame, illustrate how local biochemical support and structural stabilization can bias repair toward cartilage in rabbit models Fan et al. (2023). Stratified PCL scaffolds and printed mimetic composites add a physical layer of control by seeking to interrupt a connected mineralized path rather than simply reducing bone volume Wang et al. (2023), Yu et al. (2022).

### 3.4 Mechanical knob

The mechanical knob should first be understood as a mechanobiological and transport-control axis. The growth plate is a loaded, zonal, viscoelastic tissue rather than a passive space; reserve, proliferative, hypertrophic, and provisional ossification regions differ in cellular organization, matrix composition, water content, permeability, and compressive loading demands Kronenberg (2003), Lui et al. (2014), Lee et al. (2017). Direct measurements of growth-plate cartilage mechanics also show that material properties and strain distributions vary with anatomic location and depth, reinforcing the need for spatial rather than single-modulus benchmarks Fischenich et al. (2022). These conditions support chondrocyte survival, column formation, matrix deposition, and regulated hypertrophic progression. After injury or bar resection, matrix discontinuity, altered load transfer, abnormal confinement, and new transport paths can shift repair toward collapse, fibrosis, vascular entry, and mineralization.

The relevant mechanical variables are therefore dynamic rather than static. A construct that is too weak can collapse or permit fibrous and vascular invasion, whereas one that is too stiff, persistent, or poorly relaxing may impose nonphysiologic cues that favor hypertrophy, osteogenesis, or stress shielding. Degradation, swelling, permeability, and stress relaxation evolve during the same window in which immune resolution, vascular entry, and lineage commitment are being specified. The alginate–chitosan hydrogel study linking *in vivo* degradation rate to physeal repair outcome underscores why initial stiffness alone is insufficient; mechanics and transport must be evaluated over time Erickson et al. (2020).

Recent material studies clarify how this knob can be engineered. Polyelectrolyte-complex hydrogels with controlled mechanics altered MSC differentiation in ways relevant to growth-plate injury, while tunable GelMA–acellular-cartilage-matrix hydrogels connected scaffold viscoelasticity with Piezo1–ROCK-mediated regulation of growth-plate cartilage repair Stager et al. (2022), Shen et al. (2025). Deterministic printed frames can maintain defect geometry and constrain invasion routes, and stratified architectures better approximate anisotropic and zonal boundary conditions of the native physis Wang et al. (2023), Yu et al. (2022). Interface engineering adds a related mechanical-transport function by using compact or calcified-cartilage-like layers to resist fluid and cell migration between bone and cartilage compartments Wu et al. (2024), Wang et al. (2022), Nordberg et al. (2024a). These examples place mechanics in direct conversation with degradation, permeability, and boundary integrity, the same variables that determine whether the repair space remains column-supportive or becomes permissive to invasion and mineralized continuity.

## 4 Platform-to-knob mapping: hydrogel-centered strategies

Injectable hydrogels are particularly attractive for physeal repair because they can conform to irregular post-resection defects, provide a hydrated cartilage-like matrix, and act as local depots for cells and bioactive molecules. In the four-knob framework, they should not be considered passive fillers. Polymer chemistry, cross-linking mode, mesh size, swelling, degradation, permeability, viscoelastic relaxation, and release architecture jointly determine immune exposure, vascular entry, osteogenic takeover, and mechanotransductive cues at the defect core. Recent hydrogel advances can therefore be organized into four overlapping design families: degradation- and transport-tuned networks, extracellular-matrix-mimetic and acellular-cartilage hydrogels, staged cargo-delivery systems, and mechanically adaptive or viscoelastic gels Wang et al. (2025), Zhang et al. (2026).

Coupled degradation and transport are early determinants of hydrogel performance. Erickson and colleagues tuned alginate–chitosan formulations in a rat model and found that more rapidly degrading and more permeable formulations were associated with more cartilage-like repair, whereas slower-degrading formulations tended to favor bony tissue within the defect Erickson et al. (2020). This can be interpreted as a gating phenomenon in which the time course of permeability and degradation regulates how quickly inflammatory cells, vessels, osteogenic cells, and soluble factors access the defect core. The practical design implication is that degradation should be matched to the vulnerable inflammatory-to-osteogenic transition and reported with material-level quantities such as mass loss, swelling, permeability, and cargo-retention profiles rather than optimized only for initial injectability or cytocompatibility.

For the immune and matrix knobs, recent systems increasingly combine extracellular-matrix mimicry with immunomodulatory cargo. Exosome-loaded extracellular matrix-mimic hydrogels have been reported to suppress inflammation, promote cartilage matrix deposition, and reduce bone bridge formation after growth plate injury Guan et al. (2022). Injectable photocrosslinkable acellular cartilage matrix hydrogels loaded with exosomes extend this logic by combining cartilage-derived matrix cues, *in situ* gelation, sustained vesicle release, and chondrogenic support Si et al. (2024). These studies move the field beyond generic anti-inflammatory claims because the matrix itself can provide chondrogenic adhesion, sequestration, and degradation cues while vesicle cargo modulates inflammatory signaling; however, their functional value still depends on whether matrix preservation translates into reduced bar continuity and improved longitudinal growth.

For the vascular and signaling knobs, the strongest hydrogel studies test timing rather than simply adding a factor. Local anti-VEGF delivery reduced vascularization and bony bar formation in rats, whereas a GelMA-based rabbit system using early bevacizumab release followed by later IGF-1 support tested the principle that transient vascular delay can be paired with chondrogenic stimulation Qiang et al. (2023), Erickson et al. (2021). Other growth-plate constructs broaden this signaling logic: high-toughness adaptive hydrogels have been used to retain and present IGF-I, while PTH(1–34)-loaded PLGA microspheres combined with BMSCs, GelMA, and a PCL frame illustrate how trophic factor delivery can be integrated with mechanical stabilization Fan et al. (2023), Zhang et al. (2025c). Together, these examples connect hydrogel design to VEGF, IGF-1, and PTH/PTHrP-related pathways, but they also show why release duration, local concentration gradients, and the order of pathway engagement should be treated as design variables.

For the osteogenic knob, hydrogels can modulate lineage commitment through both matrix mechanics and molecular cargo. Injectable alginate–chitosan polyelectrolyte-complex hydrogels altered MSC differentiation tendencies relevant to growth-plate injury, supporting the idea that hydrogel composition and mechanics can bias the defect away from osteogenic conversion Stager et al. (2022). A follow-up polyelectrolyte-complex system used the same material class as a sustained siRNA delivery vehicle; MAPK14-targeting siRNA release reduced MAPK14 and downstream osteogenic gene expression in MSCs *in vitro*, although the tested *in vivo* dose did not significantly reduce bony bar formation Adhikari et al. (2024). This contrast is important because it separates pathway engagement from functional rescue and argues for reporting Runx2/ALP/OCN suppression together with microcomputed-tomography-based bar volume, bridge continuity, and growth trajectories.

For the mechanical knob, the hydrogel-specific contribution is implementation rather than another restatement of mechanotransduction theory. Dual-cross-linked hydrogels with cartilage-like extracellular matrix features show how degradation, matrix mimicry, and controllable three-dimensional mechanics can be integrated in one injectable construct Guan et al. (2023). Tunable GelMA–acellular-cartilage-matrix hydrogels connect viscoelasticity to Piezo1–ROCK-mediated growth-plate cartilage repair, while NIR-responsive curdlan/chitosan *in situ* gels add externally triggered gelation as a way to improve defect filling and local retention Shen et al. (2025), Guo et al. (2025). These examples shift the mechanical discussion from static modulus matching to time-dependent metrics, including compressive or storage modulus, stress-relaxation half-time, toughness, swelling, degradation, and permeability.

Overall, recent hydrogel studies support a more quantitative view of injectable systems: successful constructs should coordinate immune resolution, vascular delay, pathway-specific trophic support, osteogenic suppression, and time-dependent mechanics while preserving a diffusion-limited cartilage-permissive niche. Because hydrogels alone may lack sufficient boundary control in larger or irregular defects, combining injectable cores with printed frames or interfacial gating layers remains a logical translational direction that links hydrogel biology to spatial control of mineralized continuity Yu et al. (2022), Fan et al. (2023).

## 5 3D printing and structural biomimicry

Three-dimensional printing contributes a form of control that injectable materials cannot provide alone: the ability to prescribe macrogeometry, pore orientation, anisotropy, boundary features, and load-transfer paths before host repair begins Murphy and Atala (2014), Wang et al. (2021), Chung et al. (2020), Huang et al. (2025). This matters in the physis because the defect is not simply a void to be filled. A printed scaffold must keep the resection space open, resist collapse under compressive loading, and avoid creating straight vascular and osteogenic highways between epiphysis and metaphysis. Thus, pore size, filament spacing, interconnectivity, and strut orientation should be treated as biological design variables rather than neutral manufacturing parameters Liu et al. (2024), Doyle et al. (2021).

Printing also exposes a different set of tradeoffs from injectable hydrogels. Extrusion bioprinting and related approaches can position hydrogel bioinks, cells, and cues in predefined patterns, but printability requires a balance among shear thinning, shape recovery, photocrosslinking or ionic stabilization, interlayer adhesion, and longer-term mechanical integrity Liu et al. (2017), Chen et al. (2020), Yang et al. (2022), Dobrisan et al. (2024), Geevarghese et al. (2025). In physeal repair, these properties become biologically consequential. Poor filament fidelity can erase intended compartment boundaries, while excessive stabilization can reduce nutrient diffusion, stress relaxation, or matrix remodeling. The relevant question is therefore not whether a construct can be printed, but whether the printed architecture remains sufficiently faithful *in vivo* to guide invasion, preserve a cartilage-permissive core, and maintain mechanical boundary conditions during early repair.

Growth-plate-specific studies illustrate how this structural logic can be implemented without making the printed scaffold perform every biological task. Wang and colleagues used stratified polycaprolactone scaffolds with anatomy-inspired organization to approximate zonal structural features of the growth plate and reported mitigation of limb-length discrepancy and angular deformity *in vivo* Wang et al. (2023). Fan and colleagues used a printed PCL frame with hydrogel compartments and controlled factor delivery, illustrating a complementary hybrid strategy in which the printed component supplies geometric and mechanical support while the hydrogel compartment provides local biological conditioning Fan et al. (2023). Recent work on biomimetic printed scaffolds and osteochondral printing similarly emphasizes gradients, anisotropy, and compartment-specific architecture as ways to coordinate mechanical support with spatially restricted tissue ingrowth Huang et al. (2025), Liu et al. (2024).

Viewed this way, the strongest role for printing in physeal repair is not to replace hydrogels, but to give them a durable spatial grammar. Quantitative comparison should therefore include CAD-to-construct fidelity, pore and strut anisotropy, compressive modulus, degradation or creep under load, and spatial maps of host invasion, alongside bar continuity and growth outcomes. These parameters determine whether a printed implant merely occupies the defect or actively shapes the routes by which cartilage-like repair, vascular access, and mineralized consolidation unfold.

## 6 Multiphasic composites and the interfacial gating zone

A defining failure mode after physeal injury is the emergence of a rigid mineralized continuum that couples epiphysis to metaphysis and enforces growth arrest. Multiphasic constructs address this failure mode by assigning different tasks to different compartments rather than asking one material to satisfy incompatible requirements. A mechanically stable phase can preserve defect shape and peripheral integration, a hydrated or cartilage-mimetic phase can maintain a diffusion-dominated repair niche, and an interface or boundary phase can slow vascular and osteogenic access from bone-facing margins. The design target is therefore controlled compartmentalization, not maximal integration everywhere.

In this review, we use interface gating zone to denote a designed layer, gradient, or circumferential boundary feature that delays early vascular entry and osteogenic infiltration into the defect core while still permitting eventual peripheral integration. As illustrated in Figure 2, this gating zone may be implemented as a compact ring at the bone margin, a graded transition between a printed frame and hydrogel core, or a calcified-cartilage-like lamina within a multiphasic implant. Its success should be judged by whether mineralized tissue remains isolated or becomes connected. Operationally, bridge continuity can be defined as a mineralized path linking epiphysis to metaphysis across the defect, assessed by thresholded micro-CT connectivity and confirmed histologically when possible.

Yu and colleagues’ growth-plate mimetic composite is useful here primarily as a compartmentalization example rather than as another instance of printing Yu et al. (2022). In a rabbit bar resection model, the printed support maintained defect geometry, while the cartilage-mimetic hydrogel compartment provided a permissive microenvironment for non-osseous repair. By pairing tissue analyses with longitudinal limb-length and alignment measures, the study highlights a central principle for multiphasic physeal constructs: the critical endpoint is not the absence of all mineralization, but interruption of a continuous mineralized path that would tether growth.

Because direct physeal studies that deliberately reconstruct calcified-cartilage-like interfaces remain limited, osteochondral interface engineering provides transferable but imperfect guidance. Compact or mineralized interfacial layers can act as mechanical transitions and transport barriers, limiting fluid flow, cell migration, and vascular ingrowth between bone and cartilage compartments Wang et al. (2022), Nordberg et al. (2024a), Barui et al. (2023). Wu and colleagues introduced a calcified interfacial layer into a printed osteochondral scaffold and reported regeneration consistent with improved interface integrity and compartmental separation Wu et al. (2024). For physeal translation, the lesson is not to recreate adult osteochondral integration wholesale, but to adapt interface concepts toward a pediatric requirement: peripheral integration without a central epiphyseal–metaphyseal bridge, while retaining cartilage tissue-engineering criteria such as manufacturable architecture, reproducible interfaces, and functional outcome reporting Nordberg et al. (2024a), Liu et al. (2024), Doyle et al. (2021), Nordberg et al. (2024b).

Multiphasic designs therefore require a narrower optimization window than single-phase fillers. A barrier that is too permeable may simply redirect vascular invasion around the implant, whereas a barrier that is too dense or persistent may impair nutrient diffusion, remodeling, and integration with adjacent cartilage. Useful design descriptors include interface thickness, permeability, degradation rate, adhesion between phases, sealing at resection margins, and connected-component metrics for mineralized tissue. These quantities make the interfacial gating concept testable and help distinguish a true anti-bridging architecture from a construct that only appears cartilage-like at early time points.

## 7 Discussion

The knob-based organization underscores that immune, vascular, osteogenic, and mechanical processes are tightly coupled in physeal healing, which helps explain why single-factor interventions often yield inconsistent outcomes across models and time points when spatial and temporal access of vessels and osteogenic cells is not simultaneously controlled. In particular, early vascular invasion can act as a gateway that accelerates osteogenic coupling and favors bar formation, suggesting that spatiotemporal gating at the defect interface is a recurring design priority. This perspective also motivates evaluation strategies that explicitly quantify bar continuity and longitudinal growth as primary translational endpoints.

Recent studies of the human growth-plate niche, *in vitro* growth-plate engineering, growth-plate biomechanics, and cartilage-engineering translation sharpen this benchmark. Constructs should be compared not only with generic cartilage formation, but also with native physical–chemical zoning, column-supportive architecture, mechanically heterogeneous load transfer, manufacturability, reproducibility, and functional endpoints Zhang et al. (2025a), Xie et al. (2025), Fischenich et al. (2022), Nordberg et al. (2024b). For physeal applications, these considerations translate into species-aware human niche targets, zone- and depth-specific mechanical benchmarks, and continuity-sensitive anti-bridging readouts.

Consistent with this translational focus, evaluation remains a limiting factor in growth plate engineering. A minimal endpoint set for pediatric relevance integrates bar volume and continuity by microcomputed tomography, zonal organization and matrix composition by histology with immunohistochemistry, and longitudinal tracking of limb length and angular alignment Yu et al. (2022), Coleman et al. (2010). Time is an endpoint. Early suppression of vascular and osteogenic takeover within four to 6 weeks does not ensure durable growth preservation, so follow-up should span bar maturation and remodeling and report trajectories rather than terminal snapshots, with eight to 12 weeks commonly used in rabbit models Qiang et al. (2023), Fan et al. (2023).

Placing the three platform classes side by side clarifies how biomaterial design principles should be linked to signaling pathways and functional outcomes. Hydrogels provide temporal control through degradation, permeability, viscoelastic relaxation, and local release kinetics; printed scaffolds provide spatial control through geometry, anisotropy, pore architecture, and mechanical boundary conditions; and multiphasic composites provide compartmental control through interface integrity and interruption of mineralized continuity Erickson et al. (2020), Wang et al. (2023), Yu et al. (2022), Fan et al. (2023), Nordberg et al. (2024a), Liu et al. (2024). Signaling interventions such as VEGF delay, IGF-1 or PTH(1–34) support, MAPK14 suppression, and Piezo1–ROCK-related mechanotransduction should therefore be interpreted in relation to where and when the material presents them Qiang et al. (2023), Fan et al. (2023), Adhikari et al. (2024), Shen et al. (2025). A balanced framework should distinguish material feasibility, pathway engagement, tissue-level repair, and functional rescue. In practice, this means pairing release kinetics, degradation or swelling curves, architectural fidelity, interface-sealing metrics, stress-relaxation measures, and local pathway markers with continuity-sensitive imaging and limb-growth outcomes.

Interpretation also depends on model context. Age, defect size, and injury location shift repair trajectories. Larger and more central injuries preferentially form continuous bars, whereas smaller or peripheral lesions can yield partial tethering with angular deformity Coleman et al. (2010), Erickson et al. (2017). Cross-study comparison is therefore strengthened when reports include animal age, sex, and strain, the specific physis and segment, defect geometry, bar resection details, time from injury to intervention, fixation and boundary sealing strategy, and follow-up duration Yu et al. (2022). These parameters enable design choices such as degradation and release kinetics to be justified against injury-phase timelines rather than selected empirically.

Across platforms, several tensions recur. Angiogenesis supports clearance and integration yet promotes bridging when early and unrestricted. Suppressing hypertrophy and mineralization may reduce bridging but risks perturbing adjacent endochondral programs. Pathway-level interventions such as BMP antagonism suggest that dampening osteogenic takeover can curb bony repair and hypertrophic degeneration, likely within a narrow therapeutic window requiring careful localization and timing Chung et al. (2011), Su et al. (2021). Material persistence also has two sides: constructs that lose integrity too quickly can permit invasion, while constructs that persist too long or seal too completely can impede integration, nutrient exchange, and remodeling. These tradeoffs reinforce the need to validate degradation, transport, and interface behavior *in vivo* rather than infer durability from initial material properties alone Erickson et al. (2020), Nordberg et al. (2024a), Liu et al. (2024).

These points clarify how physeal biomaterials differ from osteochondral scaffolds. Adult osteochondral constructs often benefit from vascularized subchondral integration and stable coupling across cartilage and bone. Physeal constructs must preserve a diffusion-limited cartilage-permissive niche and resist early vascular and osteogenic access to the defect core. A practical trajectory is to prioritize invasion-aware architectures, including injectable viscoelastic hydrogels with tuned degradation, defect-matched printed frames with hydrogel infills, and robust boundary sealing, evaluated with longitudinal functional outcomes. More programmable control through staged delivery and explicit interface engineering can then be introduced as evidence accumulates, aligned to the four-knob framework.

## 8 Conclusion

Physeal regeneration is distinctive in that success is defined not by osseous integration but by sustained growth without a spanning mineralized bridge. Across hydrogels, deterministic printed architectures, and multiphasic constructs, advances are most consistent when platforms are used as modular controls over immune–vascular–osteogenic coupling and time-dependent mechanics, with interface gating and longitudinal outcomes treated as central design and evaluation elements.
