# Supplementary material for: Pediatric physeal repair: from immune–angiogenic–osteogenic coupling to zonal biomimetic scaffolds
Source: Front Cell Dev Biol. 2026 Jul 28;14:1802968. doi: 10.3389/fcell.2026.1802968 (PMC13457545; doi:10.3389/fcell.2026.1802968)
Supplement: Supplementary file 1 [file Table1.docx]

**Table 1. Four-knob framework: actionable design levers and suggested readouts for physeal repair constructs**

| **Controllable knob** | **Representative engineering levers** | **Suggested readouts (example time windows)** |
| --- | --- | --- |
| **Immune knob** | Material chemistry/charge; degradation byproducts; early release windows for immunomodulators (hours–days) | Local cytokines (e.g., TNF-α, IL-1β, IL-6); macrophage phenotype markers; fibrosis indices (collagen deposition) (e.g., days 3–14) |
| **Vascular knob** | Pore size/architecture; boundary sealing; transient early anti-angiogenic signaling followed by relaxation | Vessel density (CD31/endomucin); invasion depth vs time; VEGF/VEGFR expression (e.g., days 7–28) |
| **Osteogenic continuity knob** | Interface gating layers; localized mineralization inhibitors at bone margins; architectures that disrupt continuous mineralized paths | Bridge continuity on thresholded micro-CT connectivity (binary present/absent + connectivity metrics); histologic confirmation; longitudinal limb length/alignment (e.g., weeks 4–12+) |
| **Mechanical/viscoelastic knob** | Initial stiffness; stress relaxation/creep; degradation-coupled mechanics; fatigue resistance/space maintenance | Time-dependent mechanical tests (stress relaxation/creep); defect space maintenance; correlation with invasion/continuity endpoints (e.g., weeks 1–12) |
